# Closed‐Loop Deep Brain Stimulation for Essential Tremor Based on Thalamic Local Field Potentials

**DOI:** 10.1002/mds.28513

**Published:** 2021-02-06

**Authors:** Shenghong He, Fahd Baig, Abteen Mostofi, Alek Pogosyan, Jean Debarros, Alexander L. Green, Tipu Z. Aziz, Erlick Pereira, Peter Brown, Huiling Tan

**Affiliations:** ^1^ MRC Brain Network Dynamics Unit University of Oxford Oxford UK; ^2^ Nuffield Department of Clinical Neurosciences University of Oxford Oxford UK; ^3^ Neurosciences Research Centre Molecular and Clinical Sciences Research Institute, St. George's, University of London Oxford UK

**Keywords:** closed‐loop deep brain stimulation, thalamic local field potential, essential tremor, movement decoding

## Abstract

**Background:**

High‐frequency thalamic stimulation is an effective therapy for essential tremor, which mainly affects voluntary movements and/or sustained postures. However, continuous stimulation may deliver unnecessary current to the brain due to the intermittent nature of the tremor.

**Objective:**

We proposed to close the loop of thalamic stimulation by detecting tremor‐provoking movement states using local field potentials recorded from the same electrodes implanted for stimulation, so that the stimulation is only delivered when necessary.

**Methods:**

Eight patients with essential tremor participated in this study. Patient‐specific support vector machine classifiers were first trained using data recorded while the patient performed tremor‐provoking movements. Then, the trained models were applied in real‐time to detect these movements and triggered the delivery of stimulation.

**Results:**

Using the proposed method, stimulation was switched on for 80.37 ± 7.06% of the time when tremor‐evoking movements were present. In comparison, the stimulation was switched on for 12.71 ± 7.06% of the time when the patients were at rest and tremor‐free. Compared with continuous stimulation, a similar amount of tremor suppression was achieved while only delivering 36.62 ± 13.49% of the energy used in continuous stimulation.

**Conclusions:**

The results suggest that responsive thalamic stimulation for essential tremor based on tremor‐provoking movement detection can be achieved without any requirement for external sensors or additional electrocorticography strips. Further research is required to investigate whether the decoding model is stable across time and generalizable to the variety of activities patients may engage with in everyday life. © 2021 The Authors. *Movement Disorders* published by Wiley Periodicals LLC on behalf of International Parkinson and Movement Disorder Society

Essential tremor (ET) is one of the most common neurological movement disorders, in which the symptom is typically intermittent, predominantly occurring during voluntary movement and/or sustained posture.^1^, ^2^, ^3^ Severe ET can be functionally disabling and up to 50% of patients do not respond adequately to drug therapy.^4^ Continuous deep brain stimulation (DBS) is an effective therapy for ET.^5^, ^6^ However, a gradual increase of stimulation intensity is often required due to disease progression or habituation to stimulation.^7^, ^8^, ^9^ High‐intensity stimulation may induce unpleasant side effects including postural instability and speech impairment.^10^, ^11^, ^12^

Compared with conventional open‐loop DBS or thalamotomy, closed‐loop or adaptive DBS has the benefit of delivering stimulation only when symptoms are present. The reduction in delivered energy may potentially reduce side effects and possibly prolong clinical efficacy through amelioration of habituation.^13^, ^14^ Measurements from surface electromyography (EMG), wearable accelerometers, or electrocorticography (ECoG) recorded from a strip of intracranial electrodes implanted over the surface of the motor cortex have been used to detect movement states associated with tremor, or tremor itself, and to trigger the DBS.^15^, ^16^, ^17^, ^18^, ^19^ However, these existing closed‐loop DBS systems required external sensors or additional invasive instrumentation. External sensors may reduce compliance and introduce additional power demand related to communication with the pulse generator. They also potentially introduce a system vulnerability should communication be compromised or hijacked. Local field potentials (LFPs) recorded from the same DBS electrodes as used for stimulation require no additional electrodes or hardware and are likely to pick up pathological signals related to symptoms because of their location at the site of interest. Therefore, LFPs provide an alternative source of feedback control for DBS.^20^, ^21^ LFPs recorded from subthalamic nucleus (STN) have been used to develop closed‐loop DBS for patients with Parkinson's disease by detecting increased beta band power and, in acute studies, have shown superior clinical effect to conventional DBS for rigidity and bradykinesia.^22^, ^23^ In addition, Hirschmann and colleagues identified a correlation between high‐frequency oscillations in STN and parkinsonian rest tremor.^24^, ^25^ In an offline study, they demonstrated that parkinsonian rest tremor can be detected based on STN LFPs.^26^ Recently, we demonstrated that LFPs recorded from the ventral intermediate (VIM) thalamus can be used to detect voluntary movement provoking tremor and postural tremor which could be used to trigger closed‐loop DBS.^27^ However, there have not been any studies using thalamic LFPs for real‐time closed‐loop DBS for essential tremor.

In this study we designed a system for triggering closed‐loop DBS based on the real‐time detection of tremor‐provoking movements using LFPs recorded from the VIM thalamus and/or zona incerta (ZI). We tested the effectiveness of the system in eight patients who received surgery for DBS for the treatment of ET. Our results show that real‐time detection of tremor‐provoking movements can be achieved without the requirement of external sensors or additional ECoG strips, and closed‐loop DBS based on the decoding significantly reduces tremor associated with movements and sustained posture.

## Patients and Methods

### Subjects

**FIG. 1:**
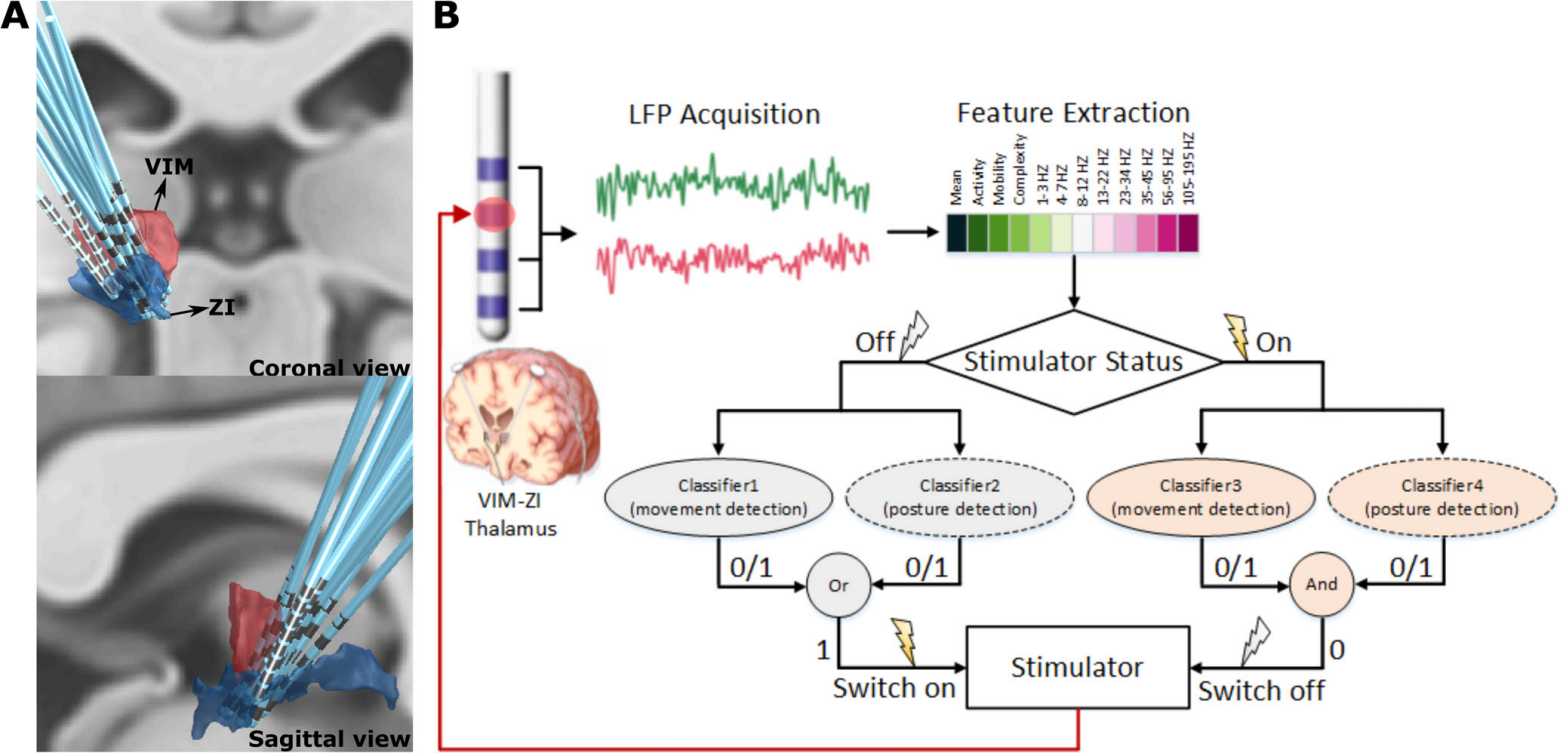
Deep brain stimulation (DBS) electrode localization and schematic of the real‐time closed‐loop DBS for essential tremor based on simultaneous measurements of ventral intermediate nucleus (VIM) and zona incerta (ZI) local field potentials (LFPs). (**A**) Three‐dimensional reconstruction in coronal view (upper image) and sagittal view (lower image) of all recorded DBS leads localized in standard space using Lead‐DBS software. Electrodes in the right hemisphere were mirrored and are shown from the left hemisphere. (**B**) Features in time and frequency domains are extracted from bipolar LFPs recorded from VIM‐ZI thalamus. If the current status of the stimulator is off and voluntary movement or tremor‐provoking posture is detected (i.e., output 1), the stimulator would be switched on. If the current status of the stimulator is on and no voluntary movement and tremor‐provoking posture is detected (i.e., output 0), the stimulator would be switched off. [Color figure can be viewed at wileyonlinelibrary.com]

Eight patients with ET (three female) who had undergone bilateral implantation of DBS electrodes participated in this study. A total of 1.5 mm spaced St. Jude Medical Infinity directional DBS leads (Abbott) were implanted in seven patients and linear Vercise DBS leads (Boston Scientific) were implanted in one patient from a second centre. In both centres the VIM motor thalamus and/or ZI were targeted for the electrode tip using classic anterior commissure posterior commissure (ACPC) stereotactic coordinates (x = +/−13, y = −4, z = 0). The clinical details of the patients and the ACPC coordinates of the most dorsal (top) and most ventral (bottom) contacts of each electrode are included in Table 1. The mean top electrode contact position in ACPC space in mm was x = 14.3 ± 2.0, y = −2.1 ± 1.7, z = 4.2 ± 2.1 (mean ± SD). Mean bottom electrode contact position was x = 11.0 ± 1.7, y = −5.3 ± 1.3, z = −1.9 ± 2.2 (mean ± SD). The Lead‐DBS MATLAB toolbox (version 2.3.2) was used to reconstruct the electrode trajectories and location of different contacts based on preoperative magnetic resonance imaging (MRI) and postoperative computed tomography (CT) scans.^28^ The electrode locations were registered and normalized into the MNI 1522009b space (Montreal Neurological Institute) using Connectomic ET target atlas,^29^, ^30^ and shown in Figure 1A. The study was approved by the local ethics committee and all patients gave informed written consent before the experiment.

**TABLE 1:** Details of patients included in the study

P	G	Age (yr)	DD (yr)	FTMTRS	DBS leads	L/R ACPC coordinates (mm)						L/R amp (V)	Predominant symptom(s) before surgery	Preoperative medication
						Top contact			Bottom contact					
						X	Y	Z	X	Y	Z			
1	F	77	21	53	Abb	14	−2.1	3.1	10	−5.5	−4.3	1.1	Tremor, gait ataxia, tremor worse on right, upper limb and voice tremor	Half Sinemet CR 125 mg at night
						13.5	−1.8	4.3	9.6	−5.1	−3.1			
2	M	61	20	56	Abb	13.1	−3	5.5	9.4	−5.1	−2.9		Tremor, dystonia, upper limb tremor and head tremor	None for tremor, previously primidone, propranolol, gabapentin, levodopa
						11.2	−4.2	4.6	9	−5.9	−0.8	3		
3	M	75	18	72	Abb	14.9	−2.2	3.5	11	−5.6	−3.4	2.5	Tremor, upper limb, lower limb and head tremor	None for tremor, previously tried primidone, clonazepam, propranolol, gabapentin, topiramate
						15.9	−0.6	3.5	11.7	−4.1	−3.5	2.0		
4	M	70	8	56	Abb	15	−2.6	5.4	10.4	−6.4	−1.4	1.8	Tremor, upper limb, with right worse than left, lower limb tremor	None for tremor, previously tried propranolol, gabapentin, topiramate, lamotrigine, primidone
						15.2	−1.8	4	10.8	−5.9	−2.6	1.8		
5	F	62	45	32	Abb	12.4	−1.8	4.4	9.5	−6.1	−3.5	2	Tremor, upper limb tremor left worse than right, voice tremor	None for tremor, previously propranolol, pregabalin, primidone
						12.2	−3.5	4.2	10	−7.6	−3.4	2		
6	M	70	5	42	Abb	15.8	0.4	3.1	11.8	−4.8	−3.2	3	Tremor, upper limb left worse than right	None for tremor, previously pregabalin, primidone, propranolol, topiramate, gabapentin
						14.6	−1.2	3.8	10.2	−6.2	−2.6	3		
7	M	67	47	54	Abb	11	−7	−2	10.5	−7.1	−3	1.5	Tremor, upper limb right worse than left, head tremor	None for tremor, previously tried propranolol, topiramate, gabapentin
						14.4	−2	4.6	13.9	−2	3.5	1.5		
8	F	65	NA	NA	Bos	18.9	0.2	6.6	15.1	−3.6	1.1	1.1	Tremor, upper limb, worse intention tremor on left	None for tremor
						17	−0.5	8.7	13.7	−4.2	2.3	1.0		
Mean		68.4	23.4	52.1		14.3	−2.1	4.2	11.0	−5.3	−1.9	1.95		
SD		5.4	15.3	11.6		2.0	1.7	2.1	1.7	1.3	2.2	0.68		

Patient 1 had gait ataxia which is sometimes seen in advanced ET. Patient 2 had an overlap between ET and dystonic tremor. Top contact indicated L4 or R4 for patients 1–7, L5 or R5 for patient 8. Bottom contact indicated L1 or R1 for all patients.

Abbreviations: P, patient; G, gender; yr, year; DD, disease duration; FTMTRS, Fahn−Tolosa−Marin Tremor Rating Scale; DBS, deep brain stimulation; L, left; R, right; ACPC, anterior commissure posterior commissure line; amp, amplitude; Abb, Abbott infinity 1.5 mm spaced leads (1−4), Abbott; Bos, Boston linear 8 contact leads (1−8), Boston Scientific; NA, not available; SD, standard deviation.

### Stimulation

For directional DBS leads, the segmented contacts of levels 2 or 3 were physically joined together to make one monopolar channel, so that we had four monopolar channels from each recorded hemisphere. A neuroBi neurostimulator (Bionics) was used to deliver monopolar stimulation to one of the monopolar DBS channels with a fixed stimulation frequency of 130 Hz, a biphasic pulse width of 60 microseconds, and an interphase gap of 20 microseconds. The electrode for the stimulation return was connected to an electrode patch attached to the shoulder of the patient, similar to the configuration used in a previous study.^22^ Prior to the experiment, the contact to stimulate and the corresponding stimulation amplitude were selected based on tremor control and side effects assessed by a clinician during progressive stepping up of stimulation amplitude. The minimal stimulation amplitude that consistently suppressed tremor when the DBS was in the ‘continuous’ mode was used for the study (more details in Table 1). Before the real‐time closed‐loop DBS test, the clinician manually changed the stimulation amplitude between 0 and the effective amplitude quickly to see if rapid changes in the stimulation amplitude induced any side effects. No participant reported long‐lasting unpleasant side effects. For the implementation of the closed‐loop DBS, only monopolar stimulation was tested, therefore the settings used during the recording might not be optimal and are likely to be different from what is used for chronic clinical stimulation.

Simultaneous bilateral stimulation was delivered in six patients, and unilateral stimulation (to the left hemisphere for patient ET1 and right hemisphere for ET2) was delivered in the other two patients since tremor was unilateral in these patients. During the experiment, the stimulation amplitude was set to the previously selected value (1–3 V; mean: 1.95 V, see Table 1 for more details) when the movement detection was positive; otherwise, the stimulation amplitude was set to 0 V.

### Data Recording

All recordings in this study were carried out 4 or 5 days after the first surgery for DBS electrodes implantation. LFPs from two adjacent contacts or two contacts neighbouring the stimulation contact were recorded in the differential bipolar mode. For the patient with linear octopolar DBS leads, three bipolar channels from four of the bottom five contacts (1−5) were recorded and analyzed. Electroencephalograms **(**EEGs) covering “Fz”, “FCz”, “Cz”, “Oz”, “C3”, “C4”, “CP3”, and “CP4” according to the standard 10–20 system were recorded in unipolar mode with common reference rejection. Bipolar EMG signals were measured from flexor carpi radialis of both arms and the masseter muscle, and acceleration measurements were acquired by triaxial accelerometers taped to the back of each hand. All these signals were simultaneously recorded using a TMSi Porti amplifier (TMS International) with a sampling rate of 2048 Hz. The reference electrode of the amplifier was connected to the chest or the wrist of the patient. The amplifier has a gain of 20 with 22 bits analogue to digital converters and resolution of 0.0715 μV per bit for both unipolar and bipolar inputs. A first‐order low‐pass filter with a −3 db point at 4.8 kHz, and a digital sinc3 filter with a cut‐off frequency of 553 Hz were implemented in the amplifier, and were applied automatically on all recorded signals. The recorded bipolar LFPs were further band‐pass filtered at 0.5–500 Hz using a forward 8th‐order Butterworth IIR band‐pass filter in MATLAB (R2018a, MathWorks).

### Closed‐Loop Control of DBS

Twelve features in time and frequency domains were extracted from recorded LFPs and fed into the pretrained classifier to detect tremor‐provoking voluntary movements and postures (Fig. 1B). Because stimulation induces changes in the neural activities and artefacts in the recording, model parameters were separately trained for different simulation status (On or Off). In addition, as our previous study suggested that the most important feature for voluntary movement and postural tremor decoding was different, in this study we trained separate patient‐specific classifiers for the voluntary movement and posture decoding.^27^ Therefore, we had four classifiers for each patient: voluntary movements and posture tremor for stimulation on and off. In the real‐time testing the right model was selected based on the status of the stimulator. If any voluntary movement or posture was detected the stimulator was switched on. If no voluntary movement and posture were detected the stimulator was switched off (Fig. 1B). Note that the stimulator was controlled automatically by the system, thus the status of the stimulator at any moment was registered by the program.

### Experimental Protocol

The experimental protocol consisted of a training session and a testing session with real‐time decoding and stimulation. During the training session each patient was asked to perform self‐paced voluntary movements: the pegboard task and/or pouring rice from one cup to another cup using the worst‐affected hand. Data were also recorded when the patients were asked to maintain tremor‐provoking postures such as raising both arms to shoulder level with flexed elbows and the fingers of both hands pointing to the centre. The voluntary movements and sustained postures were performed in blocks, with roughly 30 seconds of movements and 30 seconds of resting each block. Each patient was asked to perform 6–8 blocks of voluntary movements and 8–10 blocks of sustained postures each with and without continuous DBS, during which LFPs, EEGs, EMGs, and accelerometer measurements were simultaneously recorded. Tremor usually develops during voluntary movements or during specific postures in patients with essential tremor, so that there may be some delay between the start of movements and the onset of the tremor. In addition, the recordings were carried out only a few days after DBS electrode implantation, and tremor may not always be present in the patients tested in this study due to the postoperative stun effect. Thus, in this study we did not try to decode tremor per se. Instead, we aimed to decode the tremor‐provoking movements such as ‘voluntary movements’ which involve kinematic arm movements, and ‘posture’ which involve isometric contraction of the arms. This had the additional potential benefit that decoding might be able to anticipate tremor onset rather than follow it, so that the DBS could be switched on before the tremor was provoked by movement, allowing more time for DBS to suppress tremor.

During the real‐time decoding and stimulation test the patients were asked to repeat the voluntary movements and to maintain the tremor‐provoking postures, while the trained models were applied in real time to detect the voluntary movements and postures and to actuate the DBS. All eight patients finished the training session, which enabled us to investigate the offline decoding performance of the models in all eight patients. For the real‐time testing session, one patient (ET1) failed to participate due to some technical issues, one patient (ET3) did not participate in the testing of posture decoding, and one patient (ET7) performed movements for too short a period for the testing of voluntary movement decoding. Thus, we had in total six patients who completed the real‐time testing of posture decoding and six patients who completed the real‐time testing of voluntary movement decoding, which enabled us to evaluate the effectiveness of the proposed closed‐loop DBS system.

### Model Training

For each patient, four models were trained to decode the voluntary movement and posture with stimulation on and off based on the recorded LFPs. Specifically, the decoder output was updated at 10 Hz with an update interval of 100 milliseconds. Features were extracted based on all recorded bipolar LFPs and the label was determined based on the recorded accelerometer measurements or EMGs. The supervised training procedure for each model consisted of four steps: preprocessing, feature extraction, labeling, and classifier training (more details in Supplementary Materials S1).

### Online Testing

During online testing, the patients were asked to repeat the voluntary movements and to maintain the tremor‐provoking postures at their own pace. The classifiers with the best decoding performance during training were selected to detect the movements and postures and to actuate the DBS in real time. The detection was carried out every 100 milliseconds, resulting in a 10 Hz update rate for the DBS control. For each detection the features were extracted from the bipolar LFPs according to the same procedures during model training. The decision process for each update of the DBS control is shown in Fig. 1B.

### Offline Evaluation of the Proposed Adaptive DBS Protocol

The decoding accuracy, true‐positive rate (TPR), false‐positive rate (FPR), and false‐negative rate (FNR) of the real‐time decoding were quantified by comparing the time when the stimulation was switched on and the time when movements could be detected based on accelerometer measurements. To evaluate the effectiveness of the proposed method in suppressing postural tremor, the average power in the tremor frequency band (3–7 Hz) assessed from the accelerometer measurements recorded from the most affected hand, and the total delivered DBS energy were quantified and compared across three stimulation conditions; no DBS, adaptive DBS (A‐DBS), and continuous DBS (C‐DBS).

## Results

### Offline Decoding Performance

**FIG. 2:**
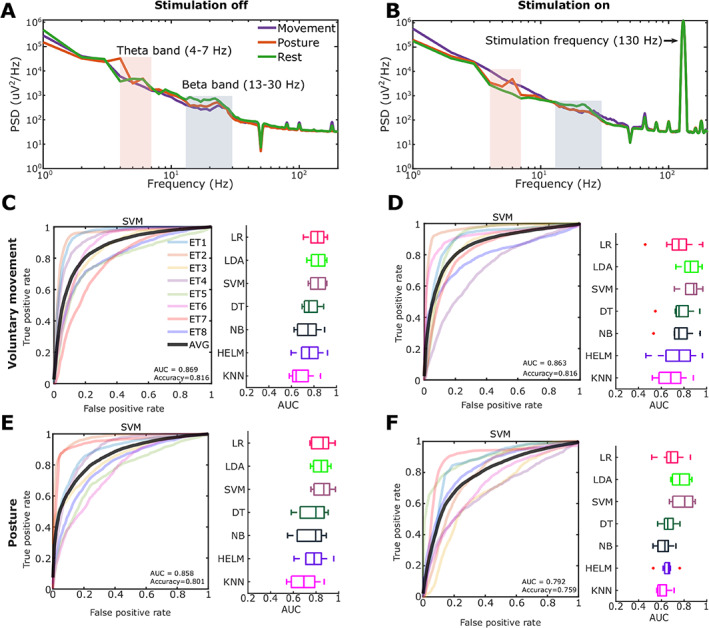
Offline decoding results using single bipolar local field potentials (LFPs) measured from contacts neighbouring the contact used for stimulation. (**A**) Average power spectra density (PSD) of thalamic LFPs during movements, posture holding, and rest when there was no stimulation. (**B**) Average PSD of thalamic LFPs during movements, posture holding, and rest when there was continuous stimulation at 130 Hz. (**C**) Decoding results for voluntary movement when there was no stimulation. (**D**) Voluntary movement when high‐frequency stimulation was switched on. (**E**) Tremor‐provoking posture when there was no stimulation. (**F**) Tremor‐provoking posture decoding when high‐frequency stimulation was switched on. Plots on the left show the receiver operating characteristic (ROC) curves of the cross‐validation with support vector machine (SVM) in different patients (different colors show results from different participants). Plots on the right show the cross‐validation area under the ROC curves (AUCs) of different classification methods. LR, logistic regression; LDA, linear discriminant analysis; DT, decision tree; NB, naïve Bayes; HELM, hierarchical extreme learning machine; KNN, k‐nearest neighbors algorithm. [Color figure can be viewed at wileyonlinelibrary.com]

Note that here we try to decode the tremor‐provoking movement states, either voluntary movements or posture holding, instead of tremor per se. The average power spectra density (PSD) showed reduced beta band activity (13–30 Hz) during voluntary movements and posture holding, compared to rest. In addition, posture holding was associated with higher theta band activities (4–7 Hz) (Fig. 2A). When stimulation was switched on (Fig. 2B), there was prominent stimulation artefact at the stimulation frequency (130 Hz) and there were also other peaks in the PSD which were related to the aliasing effect. The area under the curves (AUCs) of different classification algorithms for the detection of voluntary movement and tremor‐provoking posture with the stimulation switched on and off are shown in Figure 2C−F. Among different algorithms tested, SVM provided the best decoding performance in all conditions across all tested participants. The receiver operating characteristic (ROC) of the decoding based on SVM for all tested participants is also presented in Figure 2C−F. In the data used for training the model, the percentage of data recorded with or without movements were roughly matched: 52.55% movement versus 47.45% no movement when the stimulation was off; 52.8% movement versus 47.2% no movement when stimulation was continuously on; 57.17% tremor‐provoking posture versus 42.83% rest when stimulation was off; and 58.56% tremor‐provoking posture versus 41.44% rest when the stimulation was on. With SVM, average AUCs of 0.869 ± 0.025 (mean ± SEM), 0.863 ± 0.028, 0.858 ± 0.029, and 0.792 ± 0.033 were achieved for the decoding of voluntary movement without and with stimulation, and sustained postural without and with stimulation, respectively. These results suggest that voluntary movement and posture can be decoded using thalamic LFPs with an accuracy of around 80%, even with stimulation artefact when the stimulator was switched on. More analyses on features contributing to the decoding are presented in the Supplementary Material S1.

### Online Decoding Results

**FIG. 3:**
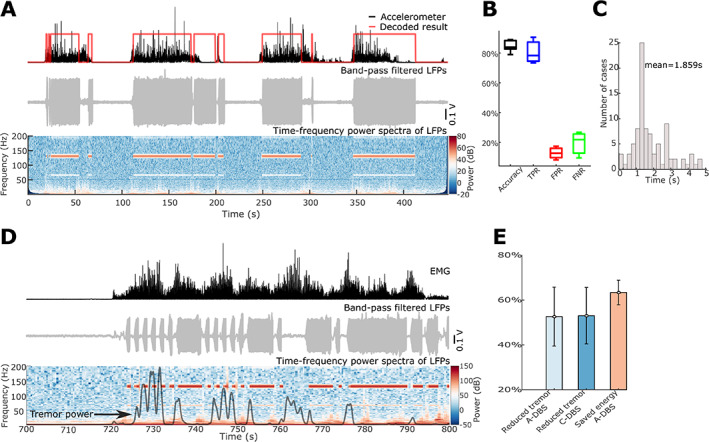
Online results of adaptive deep brain stimulation (DBS) triggered by detection of voluntary movements and/or tremor‐provoking posture. (**A**) An example of voluntary movement decoding results in patient ET4. The upper panel shows the acceleration signal with increased value indicating voluntary movements in black and the online decoding results in red with 1 and 0 for with and without movement, respectively. The middle panel shows the filtered local field potentials (LFPs) with prominent artefacts when the DBS was switched on. The bottom panel shows the power spectra of bipolar ventral intermediate nucleus‐zona incerta (VIM‐ZI) thalamic LFP signal. The red and white bands in the figure indicate stimulation artefacts at 130 Hz and subharmonic when stimulation was switched on. (**B**) Averaged accuracy, true‐positive rate (TPR), false‐positive rate (FPR), and false‐negative rate (FNR) of voluntary movement decoding during online adaptive DBS tests across six patients. (**C**) Duration distribution of all false‐negative responses (events when the voluntary movements were not detected and trigged the switching on of the DBS). (**D**) An example of tremor‐provoking posture decoding and DBS control in patient ET4. The upper panel shows the electromyography (EMG) signal in black with increased value indicating tremor‐provoking posture maintaining. The middle panel shows the filtered LFPs with prominent artefacts when the DBS was switched on. The lower panel shows the power spectra quantified using one bipolar VIM‐ZI thalamic LFP signal and the black curve shows the power of tremor frequency band activities in the accelerometer measurements. (**E**) The reduced tremor power by adaptive DBS (A‐DBS) and continuous DBS (C‐DBS) compared with no DBS, and the saved DBS energy by A‐DBS compared with C‐DBS. [Color figure can be viewed at wileyonlinelibrary.com]

During online testing, the detection of the voluntary movement and sustained posture was performed based on LFPs measured in real time to automatically drive the stimulator. The status of the DBS could be monitored according to the control signal output and the stimulation artefact in the recorded LFPs, as shown in Figure 3A. For real‐time voluntary movement decoding, the achieved average accuracy, TPR, FRP, and FNR were 84.46 ± 1.54% (mean ± SEM), 80.37 ± 2.88%, 12.71 ± 1.44%, and 19.63 ± 2.88%, respectively (Fig. 3B). This means that the DBS was switched on for 80.37 ± 2.88% of the total duration when patients were engaged in self‐paced voluntary movements; and the DBS was switched on for only 12.71 ± 1.44% of the time when the patients were at rest and free from tremor. The average duration across all false‐negative responses, that is, the episodes when DBS was switched off during voluntary movements, was 1.859 seconds (Fig. 3C). The DBS was also triggered by the detection of tremor‐provoking postures, which modulated the severity of tremor during those postures (Fig. 3D). In this acute trial, on average, A‐DBS using the proposed method suppressed the tremor by 52.62 ± 13.12% compared with no DBS, similar to the level of tremor suppression achieved by continuous DBS (53.02 ± 12.59% for C‐DBS, *t*_5_ = −0.0267, *P* = 0.9798, paired *t‐*test). However, the A‐DBS only delivered 39.62 ± 5.51% of the energy delivered during C‐DBS (Fig. 3E) in the recording session during which participants maintained tremor‐provoking posture for roughly 50% of the time. Note that a higher TPR could be achieved with a higher FPR if we reduced the decision threshold in the real‐time test. The FPR indicates the percentage of time when the DBS is switched on while not actually necessary. A higher FPR is potentially acceptable in clinical practice. Thus the TPR rates given in this study can be taken to be conservative estimates of what might be achieved during chronic stimulation.

### Detecting Tremor Using LFPs

**FIG. 4:**
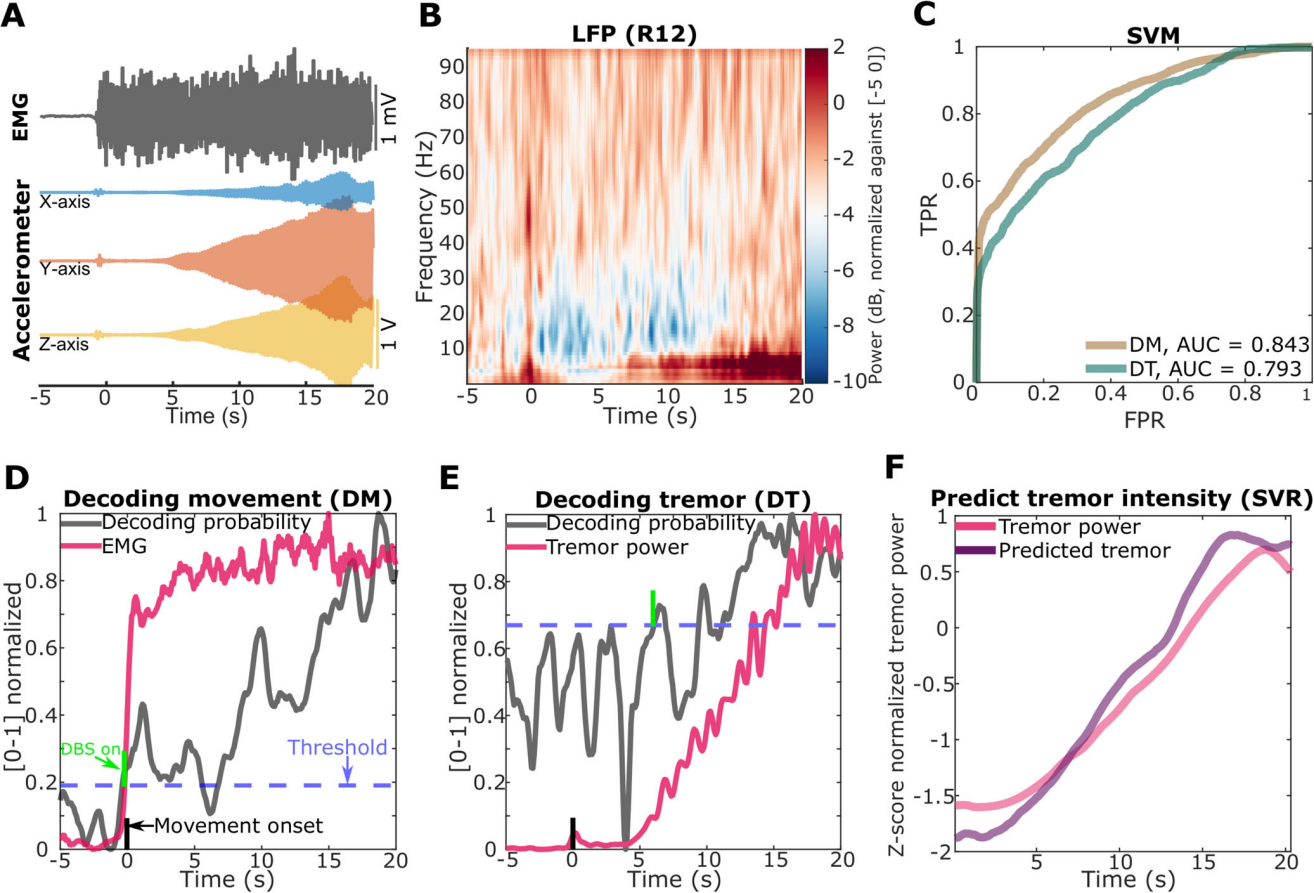
Detecting tremor using local field potentials (LFPs) (patient ET3). (**A**) An example of tremor developing (shown as increased tremor frequency band activities in accelerometer measurements) at around 5 seconds after the patient raised both arms (at t = 0, detected based on increased electromyography [EMG] activities). (**B**) Averaged power spectra across all trials of one bipolar ventral intermediate nucleus‐zona incerta (VIM‐ZI) thalamic LFP signal during posture holding, where both arms are raised at t = 0. (**C**) The receiver operating curves (ROCs) for decoding movement (DM, the brown curve) and decoding tremor (DT, the green curve) using support vector machine (SVM). (**D**) and (**E**) How decoder output changes with time (zero indicates movement onset) for decoding movement in (D) and decoding tremor in (E). The averaged decoding probabilities are shown in grey. The average EMG activity and tremor frequency power in accelerometer measurements are shown in red. The blue horizontal dashed line indicates a threshold for triggering DBS selected so that true‐positive detection rates were matched. The green vertical line indicates the average time point when the DBS would switch on using this threshold. The black vertical line indicates movement onset. (**F**) Results of tremor intensity estimation averaged across trials using single‐channel LFPs. The red and purple solid curves indicate the tremor power quantified from accelerometer and the predicted tremor power using LFP, respectively. TPR, true‐positive rate; FPR, false‐positive rate; AUC, area under the curve; SVR, support vector regression. [Color figure can be viewed at wileyonlinelibrary.com]

One patient in this cohort (ET3) showed gradually increasing tremor a few seconds after he raised his arms (Fig. 4A). Meanwhile, beta reduction and theta increment were observed in LFPs (Fig. 4B). This allowed us to test whether decoding tremor‐provoking movements would have the potential benefit of anticipating tremor as opposed to reacting to it. To do this, we compared the performance of movement decoding versus tremor decoding using SVM in the stimulation off condition. For the tremor decoding model, the training data were labeled according to the power in the tremor‐frequency band (3−7 Hz) in the accelerometer signal; whereas for the movement decoding model, the training data were labeled using the total activity of the EMG measurements. The AUC for movement decoding was higher than for tremor decoding (0.843 compared to 0.793, Fig. 4C). A threshold was then applied on the decoder output to show when DBS might be triggered. Figure 4D shows that based on movement detection, DBS would be switched on around the onset of the movement but before the development of tremor. However, if the tremor decoding model is used, the DBS would only be switched on after the development of tremor when matching true‐positive detection rates (Fig. 4E).

However, decoding of tremor has the advantage that it can be used to modulate DBS intensity according to tremor severity. We thus also tested whether we could predict the tremor intensity based on LFPs using SVM‐regression (SVR). The results showed that the tremor intensity, that is, the average power in the tremor frequency band (3–7 Hz) in the accelerometer signal, was predicted very well using single‐channel LFPs when the stimulation was switched off (Fig. 4F).

## Discussion

In this study we demonstrated that both voluntary movement and postural actions provoking tremor can be detected using thalamic LFPs despite stimulation artefact when the DBS was switched on, and verified the effectiveness of the proposed closed‐loop DBS system with real‐time stimulation testing in patients with essential tremor.

### Closed‐Loop DBS for ET Based on LFPs

Tremor in ET is intermittent, occurring when the patients are engaged in voluntary movements or maintaining certain postures, and the symptoms are further affected by factors such as mental state, fatigue, and medication state. Compared to continuous DBS or thalamotomy, closed‐loop DBS has the potential to be adaptable to changes in patients' symptoms. Here we show that closed‐loop DBS based on the detection of tremor‐provoking movements can be achieved with thalamic LFPs. The proposed methods significantly reduced tremor during voluntary movements or sustained posture while reducing the total energy delivered to the brain, and can be implemented in a fully implantable and therefore secure system without additional sensors or sensing electrodes.

### Different Decoding Algorithms

In this study the performance of several classification algorithms, which are commonly used and relatively easy to implement in real time, were tested during offline training. The algorithm with the highest decoding AUC was selected for online testing. As shown in Figure 2, linear methods including logistic regression (LR), linear discriminant analysis (LDA), and SVM achieved slightly better results than the other four methods which take into account nonlinear relationships between the features including naïve Bayes (NB), decision tree (DT), hierarchical extreme learning machine (HELM),^31^ and k‐nearest neighbors (KNN) algorithm. More complicated methods taking into account nonlinearity tend to require more data to train the model. Their relatively poorer performance could also be due to the assumption of independency between the features required by the later four classifiers. In the current application, neither the samples nor the features were independent from each other because of the overlap and coupling between different brain oscillations. Feature selection procedures such as principal component analysis (PCA) should be applied if classifiers assuming independency in the features are to be used. Some other methods have also been tested for decoding movement states based on electrophysiological signals. For example, Hidden Markov Model (HMM) has been used to detect parkinsonian rest tremor and events in gait cycles based on STN LFPs and showed promising accuracy.^26^, ^32^ A deep convolutional neural network (LFP‐Net) has also been used to recognize several different motor activities using STN LFPs.^33^ However, so far these methods have only been applied in offline analysis. Whether these more advanced algorithms could be implemented in real time to further improve decoding performance is still to be evaluated.

### The Control Algorithm

In this very first study on closed‐loop DBS for essential tremor based on real‐time decoding of tremor‐provoking movements using thalamic LFPs we have used a binary controller for the stimulator (the stimulating amplitude was either 0 or a predefined value). With this algorithm, the stimulation amplitude was dependent on a decision threshold applied to the decoder output. This led to a trade‐off between the TPR, which indicates the percentage of time when DBS is correctly switched on when it is needed, and the FPR, which indicates the percentage of time when DBS is switched on when it is actually not needed. In this study we showed that an 80/13 ratio can be achieved, but this may be a conservative estimate of what can be achieved during chronic stimulation. The best trade‐off between TPR and FPR can be different for individual patients and should be tuned accordingly in practice. In addition, the binary control algorithm can be improved by increasing/decreasing the stimulation amplitude in steps according to the detection of movement/tremor, optimizing the update rate of the DBS control and the time the DBS is kept on at each positive detection. Another approach is to get rid of the ‘decision threshold’ by modulating the stimulation amplitude continuously according to the decoder output directly, based on tremor rather than movement decoding. This would also lead to more gradual changes in the stimulation amplitude but might reduce the anticipation of tremor by DBS.

## Limitations

In our previous study, the results of offline analysis suggested that the same classifier trained on data recorded during prompted predefined movements was also able to detect other self‐paced movements, representative of those made during everyday life.^27^ The features contributing to the detection of tremor‐provoking movements defined in the current study, such as increased theta, reduced beta, and increased gamma band activities, are also those observed in different movements.^27^ However, due to the limited time we had with each patient we were not able to systematically test the generalizability of the models in real‐time decoding, or to test how stable the decoding model was across time within each patient. It would be interesting to test the A‐DBS system in a larger population with longer experimental periods especially when the patients are engaging in daily life movements. This may become feasible as devices with capacity for chronic sensing and bidirectional communication become more widely available.^13^, ^34^, ^35^ The use of chronic implanted devices would also mean that stimulation effectiveness can be tested in the absence of any stun effect. Second, we did not disassociate tremor from voluntary movements in the study; instead, we trained models to detect tremor‐provoking movements/postures. We chose this approach because tremor may not always be present in postimplantation patients due to the postoperative stun effect, and there might be some delay between the start of movements and the onset of tremor as shown in Figure 4. By detecting tremor‐provoking movement states we hoped to anticipate tremor rather than trigger stimulation after the development of tremor. Finally, it should be stressed that the comparison with conventional DBS provided in this acute study is only approximate as the tremor may already have been diminished by the postoperative stun effect, and conventional DBS was not fully optimized. The most important consideration with respect to the latter point is that conventional DBS was applied as monopolar stimulation to the same contact as adaptive DBS, without testing other contacts or stimulation modes.

In summary, we proposed a closed‐loop DBS approach for ET based on the detection of tremor‐provoking movements/postures based on LFPs recorded from the same DBS electrodes implanted for stimulation. Results from eight participants showed that tremor‐provoking movements/postures can be detected, even with high‐frequency stimulation artefact. This approach does not require external sensors or additional ECoG strips, and reduces tremor despite significantly reducing the energy delivered to the brain.

## Author Roles

(1) Research Project: A. Conception, B. Organization, C. Execution; (2) Statistical Analysis: A. Design, B. Execution, C. Review and Critique; (3) Manuscript: A. Writing of the First Draft, B. Review and Critique.

S.H.: 1B, 1C, 2A, 2B, 2C, 3A

F.B.: 1C, 2C, 3B

A.M.: 1C, 2C, 3B

A.P.: 1B, 3B

J.D.: 1C, 2C, 3B

A.L.G.: 1B

T.Z.A.: 1B

E.P.: 1B, 2C, 3B

P.B.: 1A, 1B, 2C, 3B

H.T.: 1A, 1B, 1C, 2A, 2B, 2C, 3B

## Financial Disclosures of All Authors

S.H. and H.T. report support from Medical Research Council Fund (MR/P012272/1) and Rosetrees Trust. J.D. and H.T. received research funding from The Medical and Life Sciences Translational Fund (MLSTF) and The University Challenge Seed Fund (UCSF), both from University of Oxford. A.P. and P.B. report support from Medical Research Council Fund (MC_UU_12024/1). S.H., F.B., A.P., J.D., P.B., and H.T report support from National Institute for Health Research (Oxford Biomedical Research Centre). F.B. received honoraria from Abbvie. P.B. is also a consultant form Medtronic. A.L.G. is a consultant for Abbott (Executive Advisory Board for Movement Disorders). A.M., T.Z.A., and E.P. have nothing to report.

## Supporting information


**Appendix S1.** Supporting Information.

## References

[1] Louis ED , Ottman R . Essential Tremor. The Lancet Neurology 2005;4(2):100–110. 15664542 10.1016/S1474-4422(05)00991-9

[2] Plaha P , Javed S , Agombar D , O'Farrell G , Khan S , Whone A , Gill S . Bilateral caudal zona incerta nucleus stimulation for essential tremor: outcome and quality of life. J Neurol Neurosurg Psychiatry 2011;82(8):899–904. 21285454 10.1136/jnnp.2010.222992

[3] Baizabal‐Carvallo JF , Kagnoff MN , Jimenez‐Shahed J , Fekete R , Jankovic J . The safety and efficacy of thalamic deep brain stimulation in essential tremor: 10 years and beyond. J Neurol Neurosurg Psychiatry 2014;85(5):567–572. 24096713 10.1136/jnnp-2013-304943

[4] Koller WC , Busenbark K , Miner K , Essential Tremor Study Group . The relationship of essential tremor to other movement disorders: report on 678 patients. Ann Neurol 1994;35(6):717–723. 8210229 10.1002/ana.410350613

[5] Flora ED , Perera CL , Cameron AL , Maddern GJ . Deep brain stimulation for essential tremor: a systematic review. Mov Disord 2010;25(11):1550–1559. 20623768 10.1002/mds.23195

[6] Louis ED . Treatment of medically refractory essential tremor. N Engl J Med 2016;375:792–793. 27557307 10.1056/NEJMe1606517

[7] Papavassiliou E , Rau G , Heath S , et al. Thalamic deep brain stimulation for essential tremor: relation of lead location to outcome. Neurosurgery 2004;54:1120–1129. 15113466 10.1227/01.neu.0000119329.66931.9e

[8] Pilitsis JG , Metman LV , Toleikis JR , Hughes LE , Sani SB , Bakay RA . Factors involved in long‐term efficacy of deep brain stimulation of the thalamus for essential tremor. J Neurosurg 2008;109:640–646. 18826350 10.3171/JNS/2008/109/10/0640

[9] Blomstedt P , Hariz GM , Hariz MI , Koskinen LO . Thalamic deep brain stimulation in the treatment of essential tremor: a long‐term follow‐up. Br J Neurosurg 2007;21(5):504–509. 17922323 10.1080/02688690701552278

[10] Hwynn N , Hass CJ , Zeilman P , et al. Steady or not following thalamic deep brain stimulation for essential tremor. J Neurol 2011;258(9):1643–1648. 21442464 10.1007/s00415-011-5986-0

[11] Alomar S , King NK , Tam J , Bari AA , Hamani C , Lozano AM . Speech and language adverse effects after thalamotomy and deep brain stimulation in patients with movement disorders: a meta‐analysis. Mov Disord 2017;32(1):53–63. 28124434 10.1002/mds.26924

[12] Hedera P . Emerging strategies in the management of essential tremor. Ther Adv Neurol Disord 2017;10:137–148. 28382111 10.1177/1756285616679123 PMC5367648

[13] Herron JA , Thompson MC , Brown T , Chizeck HJ , Ojemann JG , Ko AL . Chronic electrocorticography for sensing movement intention and closed‐loop deep brain stimulation with wearable sensors in an essential tremor patient. J Neurosurg 2016;127(3):580–587. 27858575 10.3171/2016.8.JNS16536

[14] Ramirez‐Zamora A , Giordano J , Gunduz A , et al. Proceedings of the seventh annual deep brain stimulation think tank: advances in neurophysiology, adaptive dbs, virtual reality, neuroethics and technology. Front Hum Neurosci 2020;14:54. 32292333 10.3389/fnhum.2020.00054 PMC7134196

[15] Graupe D , Basu I , Tuninetti D , Vannemreddy P , Slavin KV . Adaptively controlling deep brain stimulation in essential tremor patient via surface electromyography. Neurol Res 2010;32(9):899–904. 20712926 10.1179/016164110X12767786356354

[16] Basu I , Tuninetti D , Graupe D , Slavin KV. Adaptive control of deep brain stimulator for essential tremor: entropy‐based tremor prediction using surface‐EMG. In 2011 Annual International Conference of the IEEE Engineering in Medicine and Biology Society 2011: 7711–7714. 10.1109/IEMBS.2011.6091900 22256125

[17] Yamamoto T , Katayama Y , Ushiba J , et al. On‐demand control system for deep brain stimulation for treatment of intention tremor. Neuromodulation 2013;16(3):230–235. 23094990 10.1111/j.1525-1403.2012.00521.x

[18] Malekmohammadi M , Herron J , Velisar A , Blumenfeld Z , Trager MH , Chizeck HJ , Brontë‐Stewart H . Kinematic adaptive deep brain stimulation for resting tremor in Parkinson's disease. Mov Disord 2016 Mar;31(3):426–428. 26813875 10.1002/mds.26482

[19] Herron JA , Thompson MC , Brown T , Chizeck HJ , Ojemann JG , Ko AL . Cortical brain–computer interface for closed‐loop deep brain stimulation. IEEE Trans Neural Syst Rehabil Eng 2017;25(11):2180–2187. 28541211 10.1109/TNSRE.2017.2705661

[20] Little S , Brown P . What brain signals are suitable for feedback control of deep brain stimulation in Parkinson's disease? Ann N Y Acad Sci 2012;1265:9–24. 22830645 10.1111/j.1749-6632.2012.06650.x PMC3495297

[21] Priori A , Foffani G , Rossi L , Marceglia S . Adaptive deep brain stimulation (aDBS) controlled by local field potential oscillations. Exp Neurol 2013;245:77–86. 23022916 10.1016/j.expneurol.2012.09.013

[22] Little S , Pogosyan A , Neal S , et al. Adaptive deep brain stimulation in advanced Parkinson disease. Ann Neurol 2013;74:449–457. 23852650 10.1002/ana.23951 PMC3886292

[23] Rosa M , Arlotti M , Ardolino G , et al. Adaptive deep brain stimulation in a freely moving parkinsonian patient. Mov Disord 2015;30(7):1003. 25999288 10.1002/mds.26241 PMC5032989

[24] Hirschmann J , Hartmann CJ , Butz M , et al. A direct relationship between oscillatory subthalamic nucleus‐cortex coupling and rest tremor in Parkinson's disease. Brain 2013;136(12):3659–3670. 24154618 10.1093/brain/awt271

[25] Hirschmann J , Butz M , Hartmann CJ , et al. Parkinsonian rest tremor is associated with modulations of subthalamic high‐frequency oscillations. Mov Disord 2016;31(10):1551–1559. 27214766 10.1002/mds.26663

[26] Hirschmann J , Schoffelen JM , Schnitzler A , van Gerven MAJ . Parkinsonian rest tremor can be detected accurately based on neuronal oscillations recorded from the subthalamic nucleus. Clin Neurophysiol 2017;128(10):2029–2036. 28841506 10.1016/j.clinph.2017.07.419

[27] Tan H , Debarros J , He S , et al. Decoding voluntary movements and postural tremor based on thalamic LFPs as a basis for closed‐loop stimulation for essential tremor. Brain Stimul 2019;12(4):858–867. 30827864 10.1016/j.brs.2019.02.011 PMC6600875

[28] Horn A , Li N , Dembek TA , et al. Lead‐DBS v2: towards a comprehensive pipeline for deep brain stimulation imaging. Neuroimage 2019;184:293–316. 30179717 10.1016/j.neuroimage.2018.08.068 PMC6286150

[29] Avants BB , Epstein CL , Grossman M , Gee JC . Symmetric diffeomorphic image registration with cross‐correlation: evaluating automated labeling of elderly and neurodegenerative brain. Med Image Anal 2008;12(1):26–41. 17659998 10.1016/j.media.2007.06.004 PMC2276735

[30] Al‐Fatly B , Ewert S , Kübler D , Kroneberg D , Horn A , Kühn AA . Connectivity profile of thalamic deep brain stimulation to effectively treat essential tremor. Brain 2019;142(10):3086–3098. 31377766 10.1093/brain/awz236

[31] Tang J , Deng C , Huang GB . Extreme learning machine for multilayer perceptron. IEEE Trans Neural Netw Learn Syst 2015;27(4):809–821. 25966483 10.1109/TNNLS.2015.2424995

[32] Tan H , Fischer P , Shah SA , Vidaurre D , Woolrich MW , Brown P . Decoding Movement States in Stepping Cycles based on Subthalamic LFPs in Parkinsonian Patients. In 40th Annual International Conference of the IEEE Engineering in Medicine and Biology Society (EMBC) 2018;1384–1387. 10.1109/EMBC.2018.8512545 PMC6277015 30440650

[33] Golshan HM , Hebb AO , Mahoora MH . LFP‐net: a deep learning framework to recognize human behavioral activities using brain STN‐LFP signals. J Neurosci Methods 2020;335:108621. 32027889 10.1016/j.jneumeth.2020.108621

[34] Haddock A , Mitchell KT , Miller A , Ostrem JL , Chizeck HJ , Miocinovic S . Automated deep brain stimulation programming for tremor. IEEE Trans Neural Syst Rehabil Eng 2018;26:1618–1625. 29994714 10.1109/TNSRE.2018.2852222

[35] Houston B , Thompson M , Ko A , Chizeck H . A machine‐learning approach to volitional control of a closed‐loop deep brain stimulation system. J Neural Eng 2018;16(1):016004. 30444218 10.1088/1741-2552/aae67f

