# Supplementary material for: Closed‐Loop Deep Brain Stimulation for Essential Tremor Based on Thalamic Local Field Potentials
Source: Mov Disord. 2021 Feb 6;36(4):863–73. doi: 10.1002/mds.28513 (PMC7610625; doi:10.1002/mds.28513)
Supplement: Supplementary file 1 — Appendix S1. Supporting Information. [file MDS-36-863-s001.docx]

**Supplementary materials**

***Pre-processing***

The recorded bipolar LFPs were band-pass filtered at 0.5-500 Hz using a forward 8^th^-order Butterworth IIR band-pass filter. Note that the proposed adaptive-DBS was aimed to be applied in real-time, thus only those algorithms that can be implemented in real-time were used; the exception to this being the labelling used for off-line training.

***Feature extraction***

Features were calculated every 100 ms based on the filtered LFPs. For each update (sample), features including average power in eight different frequency bands, 1-3 Hz, 4-7 Hz, 8-12 Hz, 13-22 Hz, 23-34 Hz, 35-45 Hz, 56-95 Hz and 105-195 Hz, as well as the mean value and three Hjorth parameters (activity, mobility, and complexity) were calculated in ten 250-ms time windows with 150-ms overlapping between two consecutive windows for each bipolar LFP channel, resulted in 120 features (12 features per window × 10 windows).^1^ Lastly, features from all recorded bipolar LFP channels were concatenated together as the feature vector of the current update.

***Labelling***

For the training data, we gave a label of either 1 or -1 for each sample based on whether movement/sustained posture was present at the current sample. To identify the presence of movement/sustained posture, the recorded accelerometer measurements and EMGs were first high-pass filtered at 1 Hz using a forward-backward 4 order Butterworth IIR high pass filter, rectified and then smoothed using a 50-ms time window. The average values across the whole recording session of the measurements from the EMG (*T_EMG_*) and the average of all measured axis of the tri-axial accelerometer across the whole recording session (*T_ACC_*) were first calculated. For the voluntary movement, the time points with the corresponding accelerometer values larger than *αT_ACC_* were labelled as 1 and the remainder labelled as -1. For sustained posture, the time points with the corresponding EMG values larger than *αT_EMG_* were labelled as 1, and the remainder labelled as -1. Here *α* was originally set to 0.8 but was manually adjusted according to the labelling performance.

***Classifier training***

Based on the extracted features and the corresponding labels, binary classifiers were trained using several classification algorithms including logistic regression (LR), linear discriminative analysis (LDA), support vector machine (SVM), naïve Bayes (NB), decision tree (DT), hierarchical extreme learning machine (HELM)^2^, and K-nearest Neighbours (KNN). The receiver operating characteristics (ROC) curves and the area under curves (AUCs) derived from the training data using five-fold cross validation was used to evaluate the performance of different models as well as different classification algorithms. As the samples were not independent from each other, the cross validation here was applied on the original sample order rather than on randomly shuffled data to avoid information leakage.

**Features contributing to the decoding**

We then quantified the power spectra for the LFPs and accelerometer measurements used for model training, which showed beta reduction and theta band activity increment in the thalamic LFPs during movement and posture sustaining (supplementary figure 1), similar to what has been previously reported.^3^ We also investigated whether decoding relied on components in the low frequency band (<8 Hz) that might have been corrupted by movement artefact. To achieve this, we re-analysed the training data by band-pass filtering the LFPs at 8-500 Hz using a forward 8^th^-order Butterworth IIR band-pass filter to filter out low frequency activities before extracting features. We then used the features in the frequency domain above 8 Hz (8-12 Hz, 13-22 Hz, 23-34 Hz, 35-45 Hz, 56-95 Hz and 105-195 Hz) and in the time domain to train the models and to perform the cross-validation evaluation. Compared with the original results, excluding the low frequency components did not significantly reduce the decoding AUCs in all situations, suggesting that the decoding was not reliant on movement artefact (Supplementary figure 2).

Logistic regression (LR) is a statistical approach to model a binary dependent variable using the linear combination of explanatory variables (features) that are then binarized by a logistic function. Thus, we can compare the contributions of different features in decoding by comparing the average weights attributed to different features during model training. As shown in Supplementary figure 2, a negative weight in the low beta frequency band (13-22 Hz) and a positive weight in the theta frequency band (4-7 Hz) were consistently observed in all situations, indicating that the power reduction in low beta and power increment in theta were associated with positive detection. This is consistent with the beta reduction and theta band activity increase associated with voluntary movements as shown in the average movement-modulated power spectra (Supplementary Figure 1) and also consistent with our previous study.^27^

1. Hjorth B, Elema-Schönander AB. EEG analysis based on time domain properties. Electroencephalography and Clinical Neurophysiology 1970; 29: 306-310.
2. Tang J, Deng C, Huang GB. Extreme learning machine for multilayer perceptron. IEEE transactions on neural networks and learning systems 2015; 27(4): 809-821.
3. Tan H, Debarros J, He S, Pogosyan A, Aziz TZ, Huang Y, Wang S, Timmermann L, Visser-Vandewalle V, Pedrosa DJ, Green AL. Decoding voluntary movements and postural tremor based on thalamic LFPs as a basis for closed-loop stimulation for essential tremor. Brain stimulation 2019; 12(4): 858-867.

**Supplementary figures and figure legends**


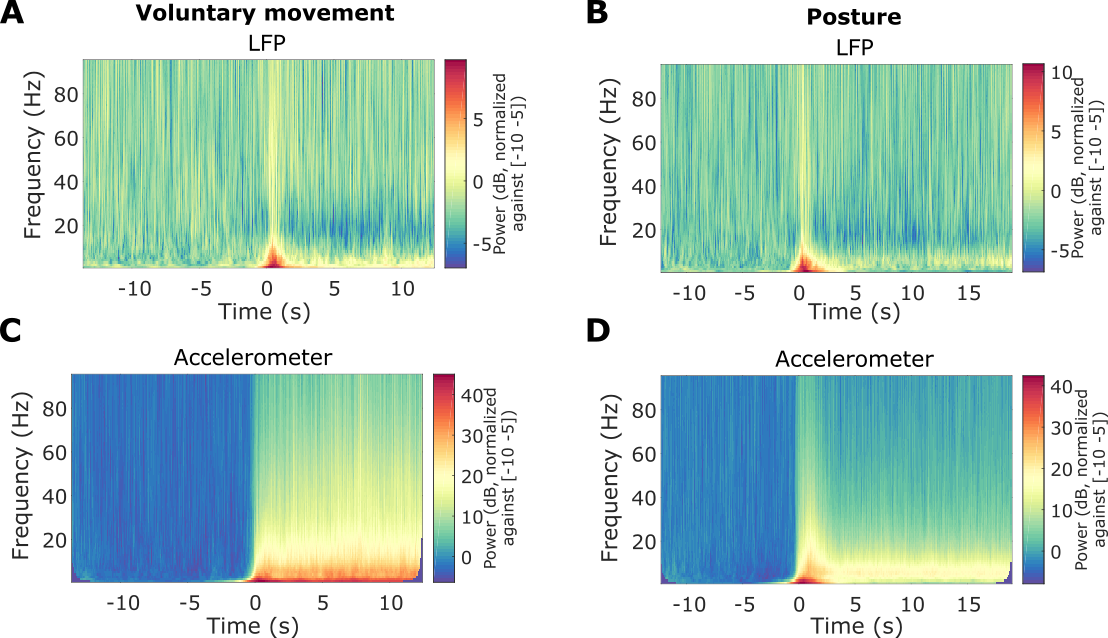


**Supplementary figure 1.** Averaged power spectra of the LFPs and accelerometer signals. Averaged power spectra of the selected LFP channel during voluntary movement (**A**) and posture holding (**B**). Averaged power spectra of z-axis accelerometer signal from the moving or tremor dominant hand during voluntary movement (**C**) and posture holding (**D**).


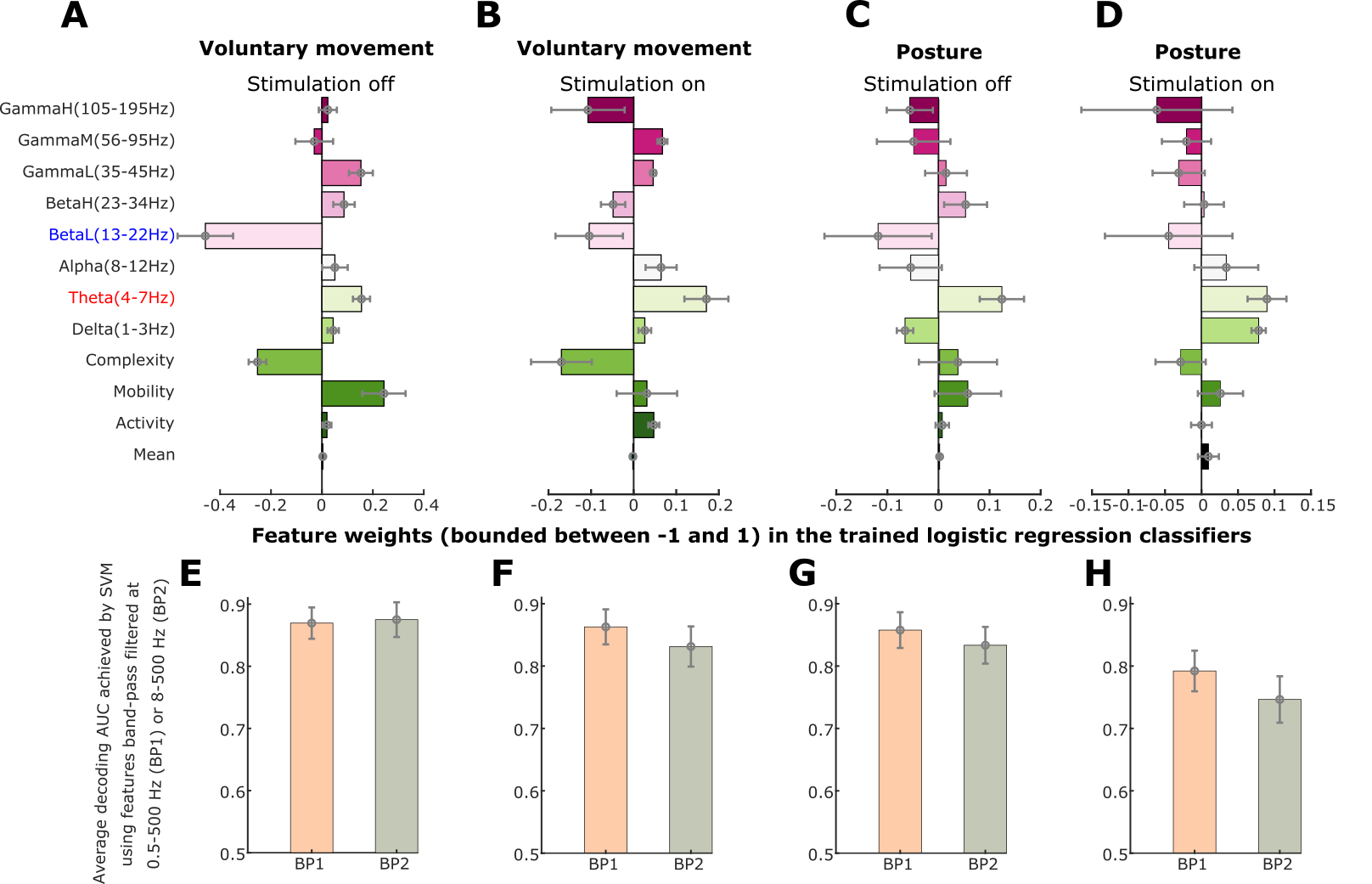


**Supplementary figure 2. Feature weights in the trained logistic regression classifiers.** (**A**) Feature weights for voluntary movement detection when there was no stimulation. (**B**) Feature weights for voluntary movement detection when high frequency stimulation was switched on. (**C**) Feature weights for tremor provoking posture detection when there was no stimulation. (**D**) Feature weights for tremor provoking posture detection when high frequency stimulation was switched on. The bar and error bar indicate the mean and SEM, respectively. The negative or positive sign indicates that a reduction or increment in the feature is associated with improved detection. No significant difference between the achieved average AUCs by SVM for voluntary movement decoding when stimulation was switched off (**E**), voluntary movement decoding when stimulation was switched on (**F**), tremor provoking posture decoding when stimulation was switched off (**G**), and tremor provoking posture decoding when stimulation was switched on (**H**) using features band-pass filtered at 0.5-500 Hz (BP1) or features band-pass filtered at 8-500 Hz (BP2, excluding features lower than 8 Hz). Thus, satisfactory decoding was achieved even if movement artefact in the LFP recordings was excluded by high-pass filtering.
